# Interictal psychosis of epilepsy: What is the role of the neurologist?

**DOI:** 10.1016/j.ebr.2024.100708

**Published:** 2024-09-02

**Authors:** Luis Pintor, Felipe Gutiérrez, Andres M. Kanner

**Affiliations:** a Consultation-Liaison Psychiatry Unit, Institute of Neurosciences, Hospital Clinic I Provincial de Barcelon, Barcelona, Catalonia, Spain; b CSMA Dreta Eixample i Camp de l ’Arpa, Fundació CPB Serveis Salut Mental, Barcelona, Catalonia, Spain; c Epilepsy Division, Department of Neurology, University of Miami, Miller School of Medicine, Miami, FL, USA

**Keywords:** Schizophrenia-like psychosis of epilepsy, Atypical antipsychotic drugs, Iatrogenic psychotic episodes, Rsperidone aripiprazole

## Abstract

Psychotic disorders are more frequent in people with epilepsy than the general population. They constitute one of the most serious psychiatric comorbidities which require an immediate psychopharmacologic intervention. Yet, access to psychiatrists is often limited or not available and the neurologists taking care of these patients are called-upon to start treatment with antipsychotic medication. The purpose of this manuscript is to provide clinicians with pragmatic psychopharmacologic strategies to treat interictal psychotic disorders in patients with epilepsy. We review the case of a 45 years-old man with a 35-year history of treatment-resistant focal epilepsy of bitemporal origin who developed a de-novo psychotic episode that began with insomnia, mood lability and agitation and evolved into paranoid delusions, auditory hallucinations and a thought disorder. The patient was diagnosed with an interictal psychotic episode and was treated with aripiprazole which resulted in significant improvement after reaching a 20 mg /day dose and allowed for the patient to be discharged home. In summary, interictal psychotic episodes of epilepsy are relatively frequent in patients with epilepsy and require of an early psychopharmacologic treatment to facilitate their remission or stabilization until mental health professionals can take over their long-term care. Compared to primary psychotic disorders, interictal psychotic episodes respond better and at lower doses of antipsychotic drugs.

## Highlights

• Interictal psychotic disorders of epilepsy are relatively frequent.
• They have a better course and prognosis than primary psychotic disorders.
• Most atypical antipsychotic drugs are safe in patients with epilepsy.
• Aripiprazole and risperidone are first line drugs in these patients.

## 1 Introduction

Psychosis of epilepsy (POE) is a term applied to a group of psychotic disorders, which can present as interictal, ictal or postictal episodes, depending on their temporal relation to the occurrence of seizures and may be also the expression of an iatrogenic process triggered by pharmacologic and /or surgical interventions [1]. The prevalence of POE has been estimated to range between 7 % and 10 % in contrast to the 0.4 % to 1 % rates of *primary* schizophreniform disorders in the general population [2], [3]. The overall evidence suggests that interictal psychosis of epilepsy (IPE), also referred as schizophrenia-like psychosis of epilepsy is believed to be six to twelve times more likely to occur in people with epilepsy (PWE) than in the general population [4]. Epilepsy and IPE have a complex and bidirectional relation, as not only patients with epilepsy are at greater risk of developing a psychotic disorder, but patients with a primary psychotic disorder are at greater risk of developing epilepsy. For example, in a population based-study conducted in Taiwan, patients with primary schizophrenia had a six-fold higher risk to develop epilepsy than controls [5], while in a population-based study using the United Kingdom’s General Practice Research Data Base, the incidence rate ratios of psychosis was 5 to 6 times higher during the three years preceding and during the three years that followed the diagnosis of epilepsy [6]. While IPE has been associated with focal epilepsy of temporal and frontal lobe origin, its etiology and pathophysiology have yet to be established [1], [2], [4].

Psychotic episodes are among the most severe psychiatric comorbidities in PWE, which under ideal circumstances should be treated by psychiatrists. However, access to mental health providers is often very limited or non-existent and neurologists may be called upon to begin pharmacotherapy to stabilize the patient’s psychotic condition until mental health professionals can take −over its management. The purpose of this manuscript is to provide clinicians with pragmatic pharmacologic strategies that can be implemented in the initial management of an IPE.

## 2 Illustrative case

The patient is a 45 years-old man with a history of treatment-resistant focal epilepsy of bitemporal lobe origin caused by a viral encephalitis at the age of 10. His seizures began at the age of 11 and have been presenting with focal aware, focal unaware and focal to bilateral tonic-clonic seizures. A presurgical evaluation revealed bilateral mesial temporal sclerosis, bitemporal independent ictal and interictal epileptiform activity, bitemporal hypometabolism and neuropsychological testing revealed verbal and visual memory deficits with greater severity of verbally mediated cognitive functions (e.g., word finding difficulty), but did not display across the board intellectual disability. For the last six months, the patient had been experiencing an average of 2 to 3 focal unaware seizures per month and one to two focal to bilateral tonic-clonic seizures on a regimen of lamotrigine, valproic acid and clobazam. He had been treated with multiple antiseizure medications (ASMs) at optimal doses and was being considered for neuromodulation therapy with responsive neurostimulation targeting both mesial temporal structures.

The patient was brought to the ER by his wife who reported a one-week history of insomnia and marked mood lability and feelings that people in the street are talking about him. In the last 24 hours, he refused to leave his room complaining that there were people in the front of his house planning to kill him. He was also heard talking aloud as if he was answering someone calling upon him (auditory hallucinations) telling him that he has special powers and not to leave his room. He barricaded himself in his room which prompted his family to take him to the hospital. Upon exam, his speech was characterized by a thought disorder consisting of loosening of associations, he was talking to himself appearing to answer to auditory hallucinations and appearing very restless. There had not been any witnessed seizures in the days preceding the onset of his psychotic symptoms, but he was admitted to the epilepsy monitoring unit to rule-out the possibility of an ictal psychotic episode, which was ruled-out on a 24-hour video-EEG monitoring study. Additional diagnostic studies included a brain MRI (under sedation) which was unchanged from previous studies and a complete blood work-up including a toxic screen, hemogram, complete metabolic panel, B12 and folic serum concentrations, VDRL, HIV, thyroid function tests, autoimmune panel including serum autoimmune antibody panel which were negative. The patient was started on aripiprazole at a dose of 5 mg /day, which was increased to 10 mg /after three days. When he reached the 10 mg /day dose, he was able to sleep through the night and he became less irritable and agitated by the fifth day, while his thought disorder remitted by the seventh day. Given the persistence of paranoid delusional thinking (“there are people next door who want to hurt me”), the dose was increased to 15 mg /day. By the time he was discharged home three weeks after his admission, the psychotic symptoms consisted of occasional delusional thinking which eventually remitted after two months on an aripiprazole dose of 20 mg /day. During this period, he continued to have focal unaware seizures every two weeks. The only adverse event of the aripiprazole was an increase in his appetite and sedation.

## 3 Discussion

*Clinical Manifestations:* The case described above represents an acute IPE in a patient with a chronic treatment-resistant focal epilepsy of bitemporal independent origin. As stated above, psychotic episodes in PWE are classified according to their temporal relation to seizure occurrence (interictal, ictal, postictal) or as an expression of an iatrogenic process to pharmacologic or surgical therapies of the seizure disorder. This patient clearly presented with an IPE, as no evidence of epileptic seizures preceding the onset of the psychotic symptoms within the previous 7 days was documented, while the ictal expression was ruled-out on video-EEG recordings. Iatrogenic causes could not be established as the patient was on a stable pharmacologic regimen of ASMs which in fact have mood stabilizing properties (e.g., lamotrigine and valproic acid). Other remote causes (structural, autoimmune encephalitis, toxic, metabolic disturbances) were excluded during his work-up.

Interictal psychotic episodes of epilepsy may be indistinguishable from primary schizophreniform disorders [7], but in a significant percentage of patients the psychiatric semiology and the course of the psychotic disorder may differ. For example, the onset of symptoms at an older age is a distinguishing feature of IPE (e.g., 45 years-old in this patient) in comparison to patients with schizophrenia whose symptoms are identified during adolescent and early adult years [8]. IPE can present as an acute or subacute episode, lasting from days or weeks and can also evolve into a chronic disorder [9], [10], lasting for months or even years. Its main clinical manifestations include delusional thinking, often with a rich affective component, auditory hallucinations and thought disorder presenting as loosening of associations, tangential and circumstantial speech. In contrast, to primary schizophrenia, IPE is characterized by a paucity or absence of negative symptoms (e.g., withdrawn attitude, lack of eye contact, blunted affect, alogia, lack of motivation, and anhedonia) [11], [12]. For example, a study using the 18-item Brief Psychiatric Rating Scale (BPRS) identified clinical differences between patients with IPE, primary schizophrenia, epilepsy without psychosis and controls [13]. This study divided the symptoms from the BPRS into three domains, negative symptoms, positive symptoms, and anxiety-depressive symptoms. The findings showed that patients with IPE had fewer negative symptoms (emotion withdrawal, conceptual disorganization, mannerism, unusual thought, motor retardation, and blunted affect) than patients with schizophrenia. These findings have been reported by other studies [12]. Another important difference is a better premorbid condition and an unlikely deterioration of personality in patients with IPE. There is a consensus of a lesser severity of psychotic symptoms and a better response to pharmacotherapy with antipsychotic drugs (APD), as seen in this patient [14]. For example, in a large prospective study carried out over one year period, patients with IPE had better response rates (27.1 %) than those with primary schizophrenia (53.8 %) and required significantly lower initial and maximal doses of APD [12].

## 4 Differential diagnosis

### 4.1 Epilepsy related

As indicated above, acute psychotic episodes can be the expression of *peri*-ictal psychotic episodes.

*Postictal psychotic episodes* (PIP) are the most frequent *peri*-ictal psychotic episodes and constitute 25 % of all psychotic episodes in PWE [15]. It is essential for all neurologists to distinguish between a PIP and an IPE. Like IPE, PIP typically occur in patients with a chronic history (>10 years) of treatment-resistant focal epilepsy, which very frequently is of bilateral independent origin. Their differences with IPE include: (i) the onset of psychotic symptoms following cluster of focal unaware and /or focal to bilateral tonic-clonic seizures; (ii) the short duration of psychotic symptoms and rapid response to low dose of antipsychotic drugs (APD) or even benzodiazepines. The delay in the onset of psychotic symptoms from the time of the last seizure (or cluster of seizures) for up to 7 days can at times preclude clinicians from recognizing the temporal relation between the seizure occurrence and psychotic symptomatology and lead to a misdiagnosis of IPE. Furthermore, 10 % to 15 % of patients with PIP may overtime develop IPE [16]; distinguishing the two is of the essence to provide the proper pharmacologic therapy. A detailed discussion of PIP can be reviewed in this issues article by Fischl and Perucca.

• *Ictal psychotic episodes* are relatively rare and have been reported in the setting of non-convulsive status epilepticus and absence status [1]. These were ruled-out in our patient with a video-EEG recording, which should always be considered in patients with new onset acute psychotic episode. Of note, PWE can experience ictal visual hallucinations, which can be distinguished from “psychotic” hallucinations when they are lateralized to a homonymous visual field and / or the patient can recognize that they are not real. However, visual hallucinations with psychotic features can also be the expression of ictal psychosis, associated with a confusional state.
• *Alternative psychosis* (or “Forced Normalization”): This is a rare condition affecting 1 % of patients with treatment-resistant epilepsy and consists of the development of psychotic and /or mood episodes following the remission of epileptic seizures triggered by pharmacologic treatment with certain ASMs (including phenytoin, primidone, carbamazepine, valproic acid, vigabatrin, benzodiazepines including clonazepam and clobazam) [17].
• *Iatrogenic:* Psychotic symptoms and episodes have been caused by several ASMs including ethosuximide, phenytoin, phenobarbital, primidone, vigabatrin, topiramate, levetiracetam, zonisamide, clobazam, lacosamide, pregabalin and perampanel [18], [19], [20], [21], [22], [23]. Acute discontinuation of benzodiazepines can also trigger acute psychotic episodes [24].

### 4.2 Psychiatric disorders

A primary psychotic disorder, such as a brief psychotic episode, schizophrenia, or schizoaffective disorder; the presence of a dissociative or conversive disorder; posttraumatic stress disorder with dissociative features; bipolar disorder, with manic episode; major depression, with psychotic presentation; as well as substance use/withdrawal, are among the differential diagnoses that need to be considered.

### 4.3 Other neurologic / medical

The list of diagnoses that need to be considered in cases of psychosis in persons with epilepsy range from oncological, infectious, autoimmune, toxic, metabolic, endocrine and degenerative disorders [1].

## 5 Screening Instruments

The Brief Psychiatric Rating Scale is an 18-item instrument that is completed by the clinician (with assistance of family members if needed) and is based on observations made over the previous two to three days [13], [25]. Each item is rated on a scale from 1 (absent) to 7 (extremely severe). Reliability on the completion of this rating scale by non-psychiatrists has not been established, however. Thus, neurologists must suspect the diagnosis on the basis of a detailed clinical history.

## 6 Pharmacologic treatment of IPE

A 2015 Cochrane review on the efficacy of antipsychotic drugs (APD) in the treatment of psychotic symptoms in epilepsy concluded that there is insufficient evidence to make any generalized recommendations [26]. Accordingly, the treatment of IPE must rely on the use of APD following the same principles applied in the management of primary psychotic disorders, taking into consideration data on their tolerability profile, pharmacodynamic and pharmacokinetic drug interactions with ASMs and their potential proconvulsant properties [1], [27] (see also the manuscript by Hamada in this special issue).

The proconvulsant properties of APDs have been reported in *patients without epilepsy* but have become an obstacle in their prescription in PWE. The actual proconvulsant properties of APDs was established in a very large study that compared the incidence of seizures during multicenter-randomized-placebo- control trials in *non-epileptic patients* between subjects randomized to an APD or placebo. Compared to placebo, clozapine had the highest seizure incidence, followed by olanzapine, and to a significantly lesser degree quetiapine [28]. The seizure incidence failed to differ between patients randomized to placebo and risperidone, aripiprazole and ziprasidone. Furthermore, all PWE are taking ASMs, which are likely to counteract the proconvulsant properties of APDs, although such antiseizure protection has only been reported in small case reports, including when using clozapine [29]. In the personal experience of one author (AMK) adjustment of the dose of ASMs has prevented an increase in seizure frequency in PWE taking clozapine, olanzapine and quetiapine (data not published).

These psychotropic drugs are divided into typical (first generation) and atypical (second generation) APD, which differ on their effect on serotonergic, dopaminergic, muscarinic and histamine receptors [1], [27] (for a full review view ref [28]). Among the first generation APD, haloperidol remains the initial choice [1], [27]. This drug, however, should only be considered if atypical APD are not available, which have displayed greater efficacy in treating negative symptoms, cognitive impairment and mood symptoms (as they have mood stabilizing properties) and their lower risk of extrapyramidal adverse events, including tardive dyskinesia [1], [27], [30]. Among the atypical APD, aripiprazole and risperidone are the first choice [1], [27], [28]. While there are six other typical and 14 atypical APDs available, their review is beyond the scope of this manuscript [30].

APDs should be started at low doses, around 25–50 % of the initial target dose, and increased in the same manner every 2–5 days. However, higher doses should be used if the severity of the psychotic disorder calls for such adjustments, as-long-as there are minimal adverse events. A previous ECG should always be requested, as most APDs have the risk of QTc prolongation [31]. The minimum effective dose of haloperidol is 2 mg/day but can be started at a dose of 4 mg to 10 mg /day for moderately severe symptoms and at doses of 10 to 15 mg /day for severe symptoms. Risperidone should be started at a dose of 2 mg/day, and 4 mg /day in the case of moderately severe to severe symptoms. Aripiprazole can be started at a dose of 5 mg to 10 mg/day and increased to 15 mg /day with a maximum dose of 20 to 30 mg /day [27], [31].

Most common adverse effects from APDs include metabolic syndrome, weight gain, sedation, extrapyramidal symptoms and hyperprolactinemia (with associated sexual dysfunction and galactorrhea) [27], [31]. Extrapyramidal symptoms and hyperprolactinemia tend to be more pronounced with typical APDs, while metabolic side effects are the hallmark of atypical APDs.

Pharmacokinetic drug interactions between ASMs and APDs can be divided into changes caused by ASMs on APDs’ clearance and vice versa [32]. The former are reflected in the increase clearance caused by carbamazepine, oxcarbazepine, phenytoin and phenobarbital of chlorpromazine, fluphenazine and haloperidol, quetiapine, aripiprazole, ziprasidone, clozapine, olanzapine and risperidone. However, phenytoin inhibits the clearance of risperidone [32]. The most relevant pharmacokinetic impact of APDs on ASMs is manifested by the inhibition of carbamazepine’s and phenytoin’s clearance by haloperidol, risperidone and quetiapine [32]. Accordingly, dose adjustments must be considered and when available, serum concentrations should be obtained.

The duration of treatment for IPE treatment duration should follow the same guidelines as the treatment for other psychotic disorders and should be established by a psychiatrist and not a neurologist.

## 7 Referral to psychiatrist

As mentioned before, psychiatric referral is warranted in all cases of IPE, as the clinical course mirrors that of other psychotic disorders. Furthermore, its frequent association with treatment-resistant epilepsy requires a multidisciplinary approach that includes the treating neurologist. Long-term management should also include a psychotherapeutic evaluation aimed at addressing medication adherence and insight through psychoeducation, providing neurocognitive rehabilitation directed at improving negative symptoms, and therapeutic approaches to treat anxiety-depressive symptoms that can be comorbid.

In this case, psychiatric referral was sought at the time of his admission and the dose of the APD was adjusted according to the course of his symptoms. Yet, in the absence of a psychiatrist on site, this treatment could have been provided by the treating neurologist following the strategies outlined above. The long-term treatment was left to the treating psychiatrist adjusting the dose requirements of APD to as low as possible.

## 8 Summary recommendations and conclusions

As illustrated in this case, neurologists should be able to begin the treatment of IPEs in PWE before a psychiatrist becomes available with the prescription of atypical APD such as risperidone or aripiprazole and haloperidol, when atypical antipsychotic drugs are not available. Furthermore, IPE tend to respond better to pharmacotherapy and at lower doses of APDs than primary psychotic episodes.

Given the potential severe risks of untreated psychotic episodes to the patient’s safety, the start of pharmacotherapy should not be delayed. The concern of proconvulsant properties of certain APDs should not be a reason to withhold treatment with APDs in PWE, particularly when using risperidone or aripiprazole. However, when APDs with proconvulsant drugs must be considered, they can be prescribed as PWE take ASMs and if necessary, their dose can be adjusted to provide a greater antiseizure protection. The long-term treatment, however, should be provided by a mental health professional in collaboration with the patient’s neurologist.

## Ethical Statement

The authors acknowledge that they have followed all the ethical rules and standards of the journal Epilepsy& Behavior Reports.

## CRediT authorship contribution statement

**Luis Pintor:** Writing – review & editing, Writing – original draft, Validation, Formal analysis, Data curation, Conceptualization. **Felipe Gutiérrez:** Writing – review & editing, Writing – original draft, Formal analysis, Data curation, Conceptualization. **Andres M. Kanner:** Writing – review & editing, Writing – original draft, Validation, Supervision, Project administration, Formal analysis, Data curation, Conceptualization.

## Declaration of competing interest

The authors declare that they have no known competing financial interests or personal relationships that could have appeared to influence the work reported in this paper.
